# Geno-Spatial Distribution of Mycobacterium Tuberculosis and Drug Resistance Profiles in Myanmar–Thai Border Area

**DOI:** 10.3390/tropicalmed5040153

**Published:** 2020-09-30

**Authors:** Htet Myat Win Maung, Prasit Palittapongarnpim, Htin Lin Aung, Komwit Surachat, Wint Wint Nyunt, Virasakdi Chongsuvivatwong

**Affiliations:** 1 National TB Programme, Department of Public Health, Ministry of Health and Sports, Naypyitaw 15011, Myanmar; htetmyetwinmaung@gmail.com; 2 Epidemiology Unit, Faculty of Medicine, Prince of Songkla University, Hat Yai, Songkhla 90110, Thailand; 3 Pornchai Matangkasombut Center for Microbial Genomics, Department of Microbiology, Faculty of Science, Mahidol University, Bangkok 10400, Thailand; prasit.pal@mahidol.ac.th; 4 Department of Microbiology and Immunology, University of Otago, Dunedin 9016, New Zealand; htin.aung@otago.ac.nz; 5 Information and Communication Technology Programme, Faculty of Science, Prince of Songkla University, Hat Yai, Songkhla 90110, Thailand; komwit.s@psu.ac.th; 6 National TB Reference Laboratory, National TB Programme, Department of Public Health, Yangon 11011, Myanmar; wintwintnyunt@gmail.com

**Keywords:** lineages of *Mycobacterium tuberculosis*, drug-resistant TB, Myanmar–Thai border

## Abstract

Worldwide, studies investigating the relationship between the lineage of *Mycobacterium tuberculosis* (MTB) across geographic areas has empowered the “End TB” program and understand transmission across national boundaries. Genomic diversity of MTB varies with geographical locations and ethnicity. Genomic diversity can also affect the emergence of drug resistance. In Myanmar, we still have limited genetic information about geographical, ethnicity, and drug resistance linkage to MTB genetic information. This study aimed to describe the geno-spatial distribution of MTB and drug resistance profiles in Myanmar–Thailand border areas. A cross-sectional study was conducted with a total of 109 sequenced isolates. The lineages of MTB and the potential associated socio-demographic, geographic and clinical factors were analyzed using Fisher’s exact tests. *p* value of statistically significance was set at < 0.05. We found that 67% of the isolates were lineage 1 (L1)/East-African-Indian (EAI) (n = 73), followed by lineage 2 (L2)/Beijing (n = 26), lineage 4 (L4)/European American (n = 6) and lineage 3 (L3)/Delhi/Central Asian (n = 4). “Gender”, “type of TB patient”, “sputum smear grading” and “streptomycin resistance” were significantly different with the lineages of MTB. Sublineages of L1, which had never been reported elsewhere in Myanmar, were detected in this study area. Moreover, both ethnicity and lineage of MTB significantly differed in distribution by patient location. Diversity of the lineage of MTB and detection of new sublineages suggested that this small area had been resided by a heterogeneous population group who actively transmitted the disease. This information on distribution of lineage of MTB can be linked in the future with those on the other side of the border to evaluate cross-border transmission.

## 1. Introduction

Myanmar and Thailand, two bordering countries, are among the top 30 high burden countries for tuberculosis (TB) [1]. The recent estimated incidence of TB in Myanmar and Thailand was 338/100,000 and 153/100,000 population, respectively [1]. Drug-resistant TB (DR-TB), impact of migration and border health issues are the important barriers for ending TB in both countries [2]. Border health is still one of the challenges in TB response especially in limited resources country such as Myanmar because of its low socio-economic level, minority ethnic groups, and migrant issues [2]. Similarly, a few recent studies from Thailand showed that there were some TB/DR-TB outbreaks in border provinces of Thailand using molecular genotyping methods [3]. With the availability of deoxyribonucleic acid (DNA) technology, genotyping has become the standard method for species and strain differentiation. The generally used molecular genotyping methods for *Mycobacterium tuberculosis* (MTB) give limited discrimination power because only a small part of the genome was examined. Whole Genome Sequencing (WGS) can overcome this limitation by providing more information about diversity of sublineages due to its very high discrimination power [4,5]. Genomic diversity affects virulence, transmissibility, host response, and the emergence of drug resistance [6,7,8]. This information on distribution of lineage of MTB across geographic areas has empowered the global TB response.

Several studies have described the distribution of lineages of MTB and their associations with geographic and ethnic background of the TB patients in other parts of Southeast Asia [9,10,11,12]. A few genotyping studies in Myanmar were previously reported [7,13,14,15]. However, WGS of *Mycobacterium tuberculosis* and DR-TB has been done only for a small number of isolates [16] and lacks geographical links to genetic information. To effectively control TB, there is a need to obtain information on drug resistance profiles of MTB in the population. Phenotypic drug susceptibility testing for the whole population is expensive. WGS information can be used to reflect the drug resistance problem, which can affect the response for ending TB. The objective of this study, therefore, was to describe the geno-spatial distribution of *Mycobacterium tuberculosis* and drug-resistant genotypes in a previously unstudied border area of Myanmar and Thailand.

## 2. Materials and Methods

### 2.1. Study Setting

In Myanmar, there are eight major national ethnic groups, such as Bamar, Kachin, Kayah, Kayin, Chin, Mon, Rakhine and Shan with another 135 minority ethnicities [17]. This study was conducted in three townships of Kayin State where many of the residents belong to the Kayin ethnicity. Kayin State is mountainous and located between latitudes 15°45′ and 19°25′ N and longitudes 96°10′ and 98°28′ E. It is bordered by Mandalay Region, Shan State and Kayah State to the north, Mon State and Bago Region to the west, and Mae Hong Son, Tak, and Kanchanaburi provinces of Thailand to the east. The three study site townships were Hpa-An, Kawkareik and Myawaddy, which all lie on the East-West Economic Corridor that links the port city of Yangon, Myanmar to South China Sea at Da Nang, Vietnam.

### 2.2. Study Design and Participants

This was a cross-sectional study conducted between March and August 2019. The patients under study were the pulmonary sputum smear-positive patients aged more than 8 years who were diagnosed and treated at one of the aforementioned township TB centers located in the township hospital compound. As culture is not routinely done in the study sites, smear-positive sputum increased on chance to have the viable MTB, which can be grown in the culture isolate after the specimen arrived at the National TB Reference Biosafety Level 3 Laboratory (NTRL) in Yangon. Xpert MTB/RIF tests were also done, but no phenotypic drug susceptibility tests (DST) were conducted following the National guideline of DRTB in Myanmar [18]. For sample size calculation, we used the finite population proportion sample size formula.

*n* = *N p*(1 − *p*)z^2^_1 − α/2_/*d*^2^(*N* − 1) + *p*(1 − *p*)z^2^_1 − α/2_ (1)

where *p* is the proportion of the most frequent lineage strain among pulmonary sputum smear-positive TB patients (0.76) based on a previous study [19], *d* is the precision (0.08), and α is the type I error rate (0.05). With these parameters, a minimum of 104 WGS TB isolates from TB patients were required. A consecutive sampling technique was used in which every subject meeting the selection criteria were selected until the required sample size was achieved. We estimated that the percentage of obtaining WGS isolates from sputum smear-positive samples was about 50%. Therefore, in order to achieve 104 WGS isolates, 200 sputum smear-positive samples were needed. These 200 smear-positive sputum samples were sent to the NTRL Laboratory in Yangon. Among them, 167 were culture growth positive and DNA was extracted. After excluding low quality DNA extracted samples, WGS of a total of 109 isolates were available in this study.

### 2.3. Samples Collection, Genomic DNA Extraction, and Whole Genome Sequencing

Two sputum samples from each patient were collected and sent to the NTRL Laboratory in Yangon. Sputum specimens were decontaminated and were then inoculated onto Lowenstein-Jensen medium for culturing according to the standard procedure. DNA was extracted using an UltraClean Microbial DNA Isolation Kit (Mo Bio Laboratories, Carlsbad, CA, USA) following the instruction manual [20]. Sequencing was performed at Massey Genome Service, New Zealand. Samples were prepared using an Illumina Nextera XT kit (Illumina, San Diego, CA, USA) as per the manufacturer’s protocol with minor modification as follows the incubation time for the tagmentation step was increased to 8 min, the PCR cycle was increased to 14 cycles and 90 µL (1.8×) of Ampure beads (Beckman Coulter, Brea, CA, USA) were used for size-selection from 400 bp to 600 bp fragments. A multiplexing step for unique identification for each MTB isolate was performed using an Illumina indices kit: Set A-D (Illumina, San Diego, CA, USA). WGS fragment sizes were quantified using a high sensitivity assay on the LabChip GX touch instrument (PerkinElmer, MA, USA). Pooling in equal molarity for sequencing was performed on a Janus robotic platform workstation (PerkinElmer, MA, USA). Prepared MTB libraries were sequenced using an Illumina MiSeq sequencer and 500 cycles kit (2 × 250 PE) (Illumina, San Diego, CA, USA). Raw sequencing reads were de-multiplexed using the Illumina RTA software (version v1.17.21.3) and SAV software (version v1.8.46) (Illumina, San Diego, CA, USA).

### 2.4. Genome Sequencing Data and Variant Calling

The system produced paired-end reads in FastQ file format. Raw reads were trimmed with Trimmomatic (v.0.3654) to remove adapter sequence and low-quality bases. Then, trimmed reads were mapped to the reference genome, *M. tuberculosis* H37Rv (Accession no. NC_000962.3) using BWA mem (v.0.7.17).

The median and average depth of sequencing were 59.72 and 47.11, respectively. 99.56% of genome was covered by paired end reads on average. Variants were called using GATK (v 3.8) by setting a minimum coverage of 10 reads, a phred score of at least 20 and a minimum allele frequency of 75% as thresholds. All single nucleotide variants (SNVs) positions were then extracted and converted to an SNV-super-matrix using an in-house Python script before being used in the phylogenetic analysis. Insertions and deletions were excluded from this study. The 109 samples of sequencing data were submitted to the European Nucleotide Archive (ENA) of EMBL-EBI mirrored in the Sequence Read Archive (SRA) database. This project was deposited in the NCBI database under “BioProject” and “BioSample” numbers, PRJNA645523 and SAMN15507652, respectively. Actual read sequences can be downloaded directly from the SRA database. (https://dataview.ncbi.nlm.nih.gov/object/PRJNA645523?reviewer=qgmhm2j6c98kdkdau8f4q54cs).

### 2.5. Phylogenetic Analysis

The SNVs of 109 isolates were used for phylogenetic tree construction. The SNVs that were present in any drug-resistant genes, mobile genetic element, phage, PE/PPE region, and non-homozygous SNVs were excluded. We analyzed the generated super-matrix with the maximum likelihood (ML) methods using RAxML (v 7.3.4). The core tree (starting tree) was generated using the BioNJ method. The tree was visualized using the FigTree program version 1.4.2. (http://tree.bio.ed.ac.uk/software/figtree/).

### 2.6. Genotyping

The genotypes were assigned based on the sublineage-specific SNPs previously listed [9].

### 2.7. Prediction of Drug Resistance

Prediction of drug resistance is based on the results from TB profiler software [21].

### 2.8. Statistical Analysis

Data from the questionnaire and record review were entered into EpiData version 3.1 (http://www.epidata.dk/) and analyzed using R version 3.6.3 (https://cran.r-project.org/). To describe the geographical distribution of major lineages of MTB and ethnicity in study townships, visualization of spatial data was done. The patients’ socio-demographic characteristics, obtained from the questionnaire and clinical characteristics obtained from medical record review, were presented descriptively. The major lineages of MTB and the potential associated socio-demographic and clinical factors, including TB drug resistance were analyzed using Fisher’s exact test. A *p* value < 0.05 was considered as statistically significant.

### 2.9. Ethics

The study was approved by the Institutional Ethics Committee of Faculty of Medicine, Prince of Songkla University, Hat Yai, Thailand (REC number 61-220-18-1) and the Ethics Review Committee of the Department of Medical Research, Ministry of Health and Sports, Myanmar (ERC number 2018−161).

## 3. Results

### 3.1. Socio-Demographic Information

Table 1 shows the socio-demographic characteristics of the TB patients by major lineages of MTB. About 50% of the patients were from Hpa-An township, the ratio of males to females was 2:1 and half were aged between 35 and 57 years. Kayin was the dominant ethnicity (47%) followed by Bamar (35%). There was no significant association between major lineages of MTB and township, age of the patients, and ethnicity. The only significantly variable was gender, where L2 was significantly more prevalent in males.

### 3.2. Genotype Information

**Figure 1:**
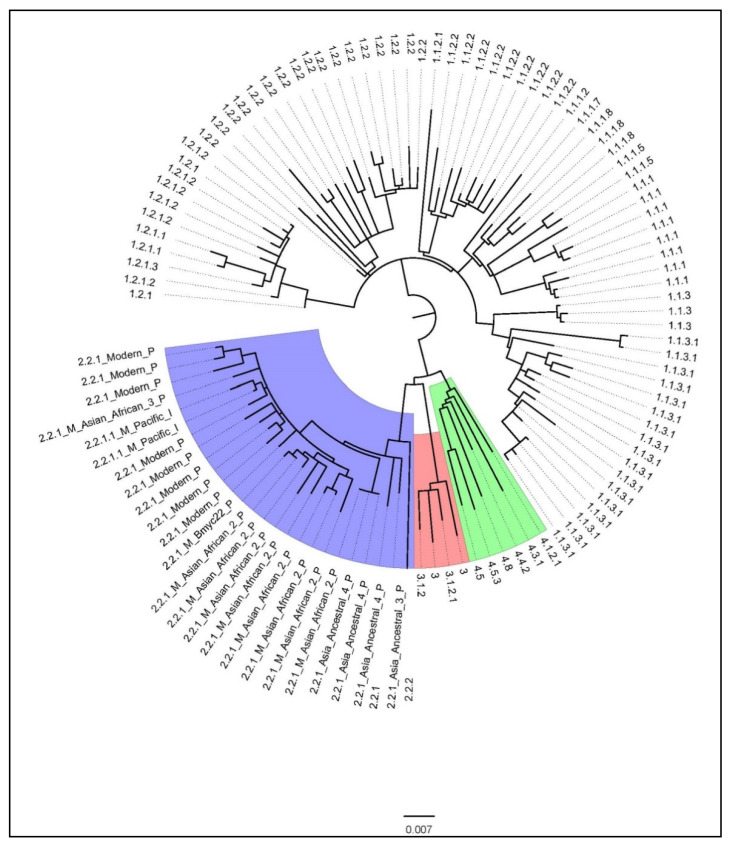
A phylogenetic tree of 109 *Mycobacterium tuberculosis* isolates from Kayin State, constructed by the maximum likelihood method.

The average coverage of the genome was 99.56%. The maximum likelihood phylogenetic tree is shown in Figure 1. About 67% of the isolates were belonged to lineage_1 (L1) or East-African-Indian (EAI) (n = 73), followed by lineage_2 (L2) or Beijing (n = 26 or 23% of isolates), lineage_4 (L4) or European American (n = 6 or 6% of isolates) and lineage_3 (L3) or Delhi/Central Asian (n = 4 or 4% of isolates). In Figure 1, the numbers and names of the sublineages are labelled at the outermost parts. The first number of the sublineage indicates the lineage. Thus, starting from the blue shaded area at 9 o’clock and moving in an anticlockwise direction shows twenty six isolates of L2, then four isolates of L3, six isolates of L4 and the remaining seventy three isolates belonged to L1. The frequency distribution of these lineages and sublineages are summarized in Table 2.

Among the most dominant lineage, L1, we found all five major sub-lineages, i.e., L1.1.1, L1.1.2, L1.1.3, L1.2.1, and L1.2.2. Unlike reports from other countries, L1.1.3 and L1.2.2 were the dominant sub-lineages contributing to about half of all L1 isolates. It should be noted that there were three L1.1.3 isolates and eight L1.1.1 isolates that could not be classified based on previously reported sublineage-specific SNPs [22]. This suggested the presence of new sublineages of L1 in Kayin state.

All L2 isolates belonged to L2.2 or the Beijing family. The majority (20/26) were Modern Beijing strains, with most belonging to the L2.2.1. Asian African 2 sublineage. There was an isolate belonging to the L2.2.1.Bmyc22 sublineage, which was previously reported only from Thailand [9]. Most of the Ancestral Beijing isolates belonged to L2.2.1. Ancestral 4, a recently described sublineage common in other Tibeto-Burman speaking tribes, Akha, and Lahu. L2.2.1. Ancestral 4 is the Ancestral Beijing strains that is most genetically related to the Modern Beijing strains, with the presence of G1286766C (mutT2 codon 58) but not the ogt12 mutation [9]. The former was previously considered as a specific barcoding SNP marker of Modern Beijing strains. L3 and L4 strains were found in a small portion in this study.

### 3.3. Geno-Spatial Analysis

**Figure 2:**
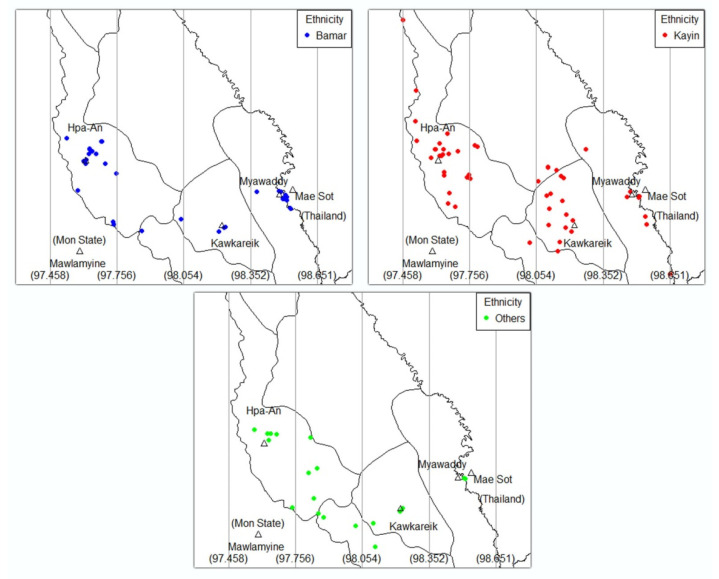
Geographic distribution of *Mycobacterium tuberculosis* patients by ethnicity.

**Figure 3:**
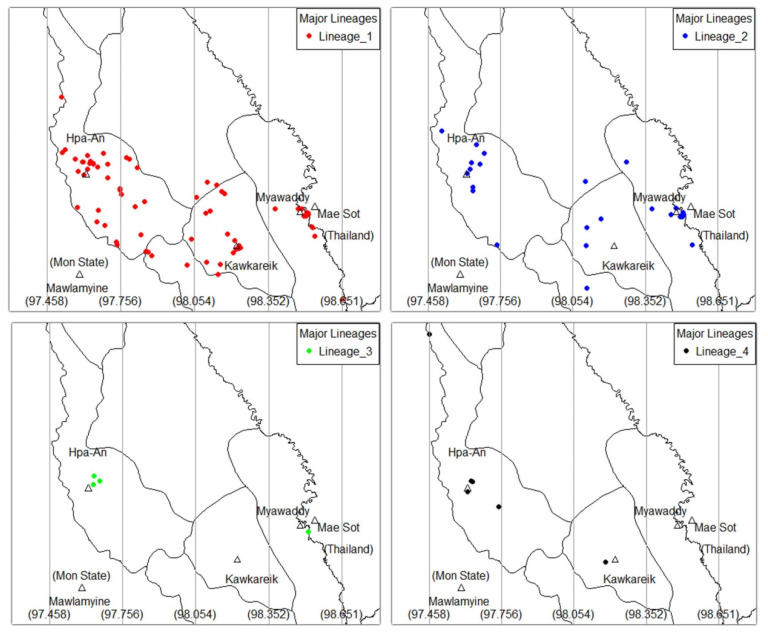
Geographic distribution major lineages of *Mycobacterium tuberculosis*.

Figure 2 and Figure 3 illustrate maps of the patients’ residence by ethnicity and major lineages of MTB, respectively. The range of longitudinal coordinates was equally divided into four zones. The cities are in zone 1 (Hpa-An), zone 3 (Kawkareik) and zone 4 (Myawaddy). Statistics of these distributions are summarized in Table 3. Both ethnicity and major lineages of MTB significantly differed in distribution by zone. Bamar were mainly living in zones 1 and 4 whereas Kayin were mainly in zones 1 and 3. Kayin, the majority ethnic group in this study, were scattered in zones where the cities are situated but most of them lived outside the city. Most Bamar patients lived in Hpa-An and Myawaddy city. Other ethnic groups were scattered in rural areas of zone 2 and few in all three cities. Zone 1 had all type of major lineages of MTB. Zone 3 and 4 showed L1 and L2 dominant and zone 2 had only the L1 lineage.

### 3.4. Clinical Characteristics

Detail information of the clinical characteristics of the study patients and the distribution of major lineages is shown in Table 4. Sputum grading was the only variable significantly different with the major lineages of MTB. Most of L1, L3, and L4 lineages showed high smear grading “2+ and 3+” and few were smear grading “1+”. However, about 43% of L2 showed the smear grading “scanty and 1+”. The variable type of TB patient was marginally significant with the distribution of major lineages of MTB. About a quarter of L2 patients were retreated TB patients. However, few L1 patients were retreated TB patients and there were no retreated L3 and L4 patients. The rest of the clinical characteristics had no significant difference with major lineages.

### 3.5. Drug Resistance of TB Drug Information

When we analyzed the drug resistance of TB drugs, we excluded the isolates that were less than 30 × depth and 95% genome completion coverage and so 63 isolates were analyzed to detect the drug-resistant profiles in this study. Table 5 summarized the association between drug susceptibility and major lineages of MTB. Out of 63 isolates, 19 (30%) had at least one anti-TB drugs resistance mutations. Most common resistance was found against Isoniazid and followed by Ethionamide and Streptomycin. Among TB drugs, Streptomycin resistance was the only one associated with major lineages of MTB.

Table 6 displays detailed information on identified variants associated with drug resistance in the genomes of the 19 clinical *Mycobacterium tuberculosis* complex isolates. Two of the Isoniazid-resistant isolates were also resistant to Rifampicin and Pyrazinamide, Ethambutol and Streptomycin. These two MDR-TB patients tested positive for Rifampincin resistance on Xpert MTB/RIF, while the Xpert MTB/RIF tests of the other isolates showed no Rifampicin resistance. Both MDR-TB isolates belonged to the L2 lineage. Among eight poly-DRTB isolates, four were L1 lineage and another four were L2 lineage. Six out of eight poly-DRTB isolates had resistance to Isoniazid. The remaining nine were mono drug-resistant tuberculosis. There were no pre-extensively drug-resistant tuberculosis (Pre-XDRTB) and extensively drug-resistant tuberculosis (XDRTB) cases in this study. Both RIF-resistant isolates showed resistance-determinant mutations in *rpoB* gene, either Ser450Leu or His445Asp. Among the INH-resistant strains, 10/13 (77%) had a Ser315Thr mutation in the *katG* gene, 2/13 (15%) showed a mutation in the *fabG1* gene and the other one had a mutation in the *ahpC* gene. Details of drug-resistant mutation points are shown in Table 6.

**Figure 4:**
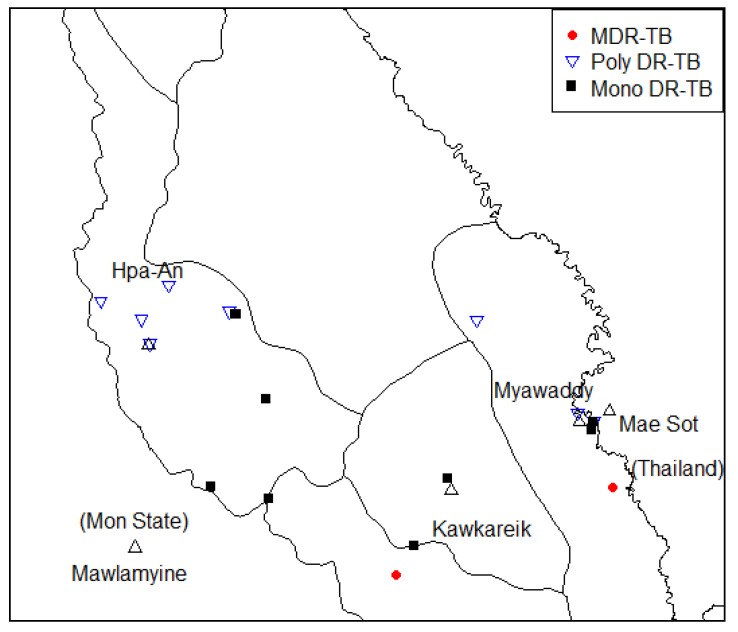
Geographic distribution of drug-resistant TB patients.

Figure 4 shows the geographical distribution of drug-resistant TB patients in this study. Among the 19 drug-resistant TB patients, one MDR-TB patient lived near the Myanmar–Thai border line. The other lived in land and the two were approximately 49.8 km away from each other.

## 4. Discussion

The *Mycobacterium tuberculosis* population in the study area was genetically diverse with the total of 4 lineages and at least 30 sublineages identified among 109 study isolates. Around two-thirds were lineage 1 and approximately a quarter was lineage 2. Among the socio-demographic characteristics, gender was the only variable significantly associated with major lineages. Both ethnicity and major lineages differed in their distribution by longitudinal zone of patients’ locations. Some clinical characteristics such as “type of TB patient”, and “sputum smear grading were significant difference with the lineages of MTB. Moreover, we found an association between drug resistance and major lineages of MTB and identified 19 drug-resistant isolates in this study. However, there was no evidence of an association between lineage and patients’ ethnicity. There was also no specific geographical pattern on drug-resistant isolates.

The descriptive statistics on distribution of major lineages were significantly different between male and female patients. The proportion of males was 2.2 times higher than females overall and significantly higher among L2 lineage (88% of L2 isolates) and the finding was contrasted with other studies from China [23,24]. Similar to WHO data and another study from Myanmar, about half of the patients were from the most active and productive age group [1,2]. Another study also found that patients with lineage 1 were older than those with other lineages and our study also showed that the mean age of patients with lineage 1 was higher than those with lineage 2 and lineage 4. However, the difference was not statistically significant [9]. A previous study in Thailand showed that some major lineages and sublineages such as the Ancestral_4 strain was associated with ethnic groups that speak Tibeto-Burman language family such as Akha and Lahu tribes, which were originally from Yunnan, China [25,26] compared to Thai ethnic group [9]. Although there was no significant association between the major lineages and patients’ ethnicity, here we identify the Ancestral_4 strain in both Bamar and Kayin ethnic groups, both of which also speak the Tibeto-Burman language family. This suggests that the sublineage may be associated with several ethnic groups that speak the language family. Further studies on the ethnic association may provide information the origin of the sublineage.

Table 7 compares the distribution of lineage of MTB from our study with that from other studies [9,11,19,27]. L1 lineage was the most dominant in our and the Philippines study but in detail, the sublineage pattern was different [11]. L2 lineage was very prominent in a study from Yangon Region, Myanmar [19]. This difference between lineage distribution of our study and the Yangon study suggests that transmission of TB in border areas has relatively little communication with the Yangon Region. It also explains the relatively low prevalence of multiple drug-resistant TB compared to Yangon Region. With similar findings from previous studies, the proportion of L3 and L4 lineages were quite low, compared with L1 and L2 lineages [7,19].

By using a combination of spatial analysis and geographic information system (GIS), we could visualize the distribution of major lineages of MTB and ethnicity. All of the zones showed significant L1 lineage dominant but in zone (4) where the number of L1 and L2 lineages were roughly equal. L2 lineage was more prevalent on the Myanmar–Thai border than central region, and this finding was consistent with a study from Thailand, which showed that the L2 lineage was dominant in a border province [3]. Moreover, all zones showed that the Kayin ethnicity was dominant; except for zone (4), which had a predominance of the Bamar ethnicity. This may be due to the location of main residential areas of Myawaddy city where there are many Bamar cross-border migrant workers. Furthermore, most people of Bamar ethnicity live near the main cities but most Kayin and other minority ethnicities are scattered in rural areas. Further studies are needed to better understand phylo-geographic variation of TB disease, and explore local risk factors that may be responsible for the observed pattern.

There were only two clinical features significantly associated with the lineage of MTB in our study: type of TB patient and sputum grading. We found that the L2 lineage had a high percentage of retreated cases, consistent with previous reports that showed this lineage to have a higher relapse rate and an association with drug resistance [9,28,29]. However, our finding of higher sputum grading in L1 lineage was not consistent with the finding of no association in South Africa study and higher grading of L2 in Sri Lanka study [30,31].

The anti-TB drug resistance burden predicted from WGS here was quite similar to that reported in previous studies in Myanmar [28,32]. Many of the studies showed that L2 lineages had significant association with DRTB and our studies also highlighted that they were associated with streptomycin resistance and both MDR-TB isolates were L2 lineages [16,19]. In addition, the study also highlighted that none of the strains in this study were predicted to be pre-XDRTB and XDRTB. Rifampicin and Isoniazid, the two most powerful anti-TB drugs, were resistant due to mutations in the *rpoB* and *KatG* genes respectively, and the finding was similar to previously observed study in Myanmar [16].

In Myanmar, National TB Programme stills cannot introduce first-line and second-line DST in routine diagnosis for all registered TB patients and those tests are mainly focus on the registered DRTB patients only. However, according to the National guideline of DRTB in Myanmar, all of the registered smear-positive TB patients need to be tested for Xpert MTB/RIF with free of charge [18] and we have the results of Xpert MTB/RIF in our system. The Xpert MTB/RIF results of the two RIF resistance isolates were consistent with our study. Our study can also highlight that if we use routine diagnosis flow, we can detect only two RIF resistance patients, but if we could use WGS in future, we can detect the additional drug resistance.

In this study, we could combine the genetic results from the whole genome sequencing and geographical data and patients’ background and clinical characteristics. The study was the first in Kayin ethnic areas near Myanmar-Thai border. The limitation of this study was on its small number of isolates. The local health services and the transportation conditions did not allow us to recover all the potential isolates collected in the field. Generalization of the results to the whole community in this area must be made with caution.

## 5. Conclusions

Non-significant ethnic differential but significant geographic differential of lineage of MTB in this study area may suggest relatively well-mixed ethnicity. It may also suggest that an area focus is more justifiable than an ethnic focus in local ending TB. Non-clustered drug-resistant MTB genotypes and relatively few MDR-TB cases compared to the national scale indicate an opportunity to prevent MDR-TB spread in this locality.

## Figures and Tables

**Table 1:** The Socio-Demographic Characteristics of tuberculosis (TB) Patients and Major Lineages of *Mycobacterium tuberculosis* (MTB).

	L1 n (%)	L2 n (%)	L3 n (%)	L4 n (%)	Total n (%)	*p* Value *
Total	73	26	4	6	109	
Township						0.1
Hpa-An	35 (48)	10 (38)	3 (75)	5 (83)	53 (49)	
Kawkareik	24 (33)	5 (19)	0 (0)	1 (17)	30 (28)	
Myawaddy	14 (19)	11 (42)	1 (25)	0 (0)	26 (24)	
Gender						0.02
Male	47 (64)	23 (88)	1 (25)	4 (67)	75 (69)	
Female	26 (36)	3 (12)	3 (75)	2 (33)	34 (31)	
Age						0.07
Mean (SD)	46 (15)	43 (13)	58 (7)	36 (13)	45 (14)	
Age group (years)						0.15
<35	17 (23)	8 (31)	0 (0)	3 (50)	28 (26)	
35−57	35 (48)	14 (54)	1 (25)	3 (50)	53 (49)	
≥57	21 (29)	4 (15)	3 (75)	0 (0)	28 (26)	
Ethnicity						0.43
Bamar	23 (32)	11 (42)	2 (50)	2 (33)	38 (35)	
Kayin	33 (45)	13 (50)	1 (25)	4 (67)	51 (47)	
Others	17 (23)	2 (8)	1 (25)	0 (0)	20 (18)	

L: lineage; SD: standard deviation; * Fisher’s exact test.

**Table 2:** Frequency Distribution of Major Lineages and Sublineages.

Major Lineage	Sublineage	n	Total (109)
L1 (EAI)	L1.1.1	8	73
	L1.1.1.2	1	
	L1.1.1.5	2	
	L1.1.1.7	1	
	L1.1.1.8	3	
	L1.1.2.1	1	
	L1.1.2.2	10	
	L1.1.3	3	
	L1.1.3.1	17	
	L1.2.1	1	
	L1.2.1.1	2	
	L1.2.1.2	6	
	L1.2.1.3	1	
	L1.2.2	17	
L2 (Beijing)	L2.2.1.1_M_Pacific	2	26
	L2.2.1_Asia_Ancestral_2	1	
	L2.2.1_Asia_Ancestral_3	1	
	L2.2.1_Asia_Ancestral_4	3	
	L2.2.1_M_Asian_African_2	8	
	L2.2.1_M_Asian_African_3	1	
	L2.2.1_M_Bmyc22	1	
	L2.2.1_Modern	8	
	L2.2.2	1	
L3 (Delhi/CAS)	L3	2	4
	L3.1.2	1	
	L3.1.2.1	1	
L4 (Euro-American)	L4.1.2	1	6
	L4.3	1	
	L4.4	1	
	L4.5	1	
	L4.5.3	1	
	L4.8	1	

L: Lineage; EAI: East-African-Indian; Delhi/CAS: Delhi/Central Asian.

**Table 3:** Distribution of Patient Ethnicity and Major Lineages of *Mycobacterium tuberculosis*.

	Zone1 (97.458–97.646)	Zone2 (97.646–97.854)	Zone3 (97.854–98.244)	Zone4 (98.244–98.650)	Total	*p* Value *
Total	45	14	25	25	109	
Ethnicity group						<0.001
Bamar	17 (37.8)	2 (14.3)	3 (12.0)	16 (64.0)	38 (34.9)	
Kayin	22 (48.9)	4 (28.6)	18 (72.0)	7 (28.0)	51 (46.8)	
Others	6 (13.3)	8 (57.1)	4 (16.0)	2 (8.0)	20 (18.3)	
Major Linage						0.047
L1	27 (60.0)	14 (100)	18 (72.0)	14 (56.0)	73 (67.0)	
L2	10 (22.2)	0 (0)	6 (24.0)	10 (40.0)	26 (23.9)	
L3	3 (6.7)	0 (0)	0 (0)	1 (4.0)	4 (3.7)	
L4	5 (11.1)	0 (0)	1 (4.0)	0 (0)	6 (5.5)	

* Fisher’s exact test.

**Table 4:** The Clinical Characteristics of TB Patients and Major Lineages of *Mycobacterium tuberculosis.*

	L1 n (%)	L2 n (%)	L3 n (%)	L4 n (%)	Total n (%)	*p* Value *
Total	73	26	4	6	109	
Blood in sputum						0.9
No	55 (75)	20 (77)	4 (100)	5 (83)	84 (77)	
Yes	18 (25)	6 (23)	0 (0)	1 (17)	25 (23)	
Fever > 2 weeks						0.17
No	34 (47)	18 (69)	3 (75)	4 (67)	59 (54)	
Yes	39 (53)	8 (31)	1 (25)	2 (33)	50 (46)	
Unexplained weight loss						0.35
No	14 (19)	5 (19)	2 (50)	2 (33)	23 (21)	
Yes	59 (81)	21 (81)	2 (50)	4 (67)	86 (79)	
Chest Pain						0.35
No	26 (36)	9 (35)	3 (75)	1 (17)	39 (36)	
Yes	47 (64)	17 (65)	1 (25)	5 (83)	70 (64)	
Severity of cough						0.05
Mild	23 (32)	8 (31)	2 (50)	3 (50)	36 (33)	
Moderate	41 (56)	10 (38)	0 (0)	3 (50)	54 (50)	
Severe	9 (12)	8 (31)	2 (50)	0 (0)	19 (17)	
HIV status						0.46
Positive	3 (4)	1 (4)	0 (0)	1 (17)	5 (5)	
Negative	70 (96)	25 (96)	4 (100)	5 (83)	104 (95)	
Type of TB Patient						0.04
New	70 (96)	20 (77)	4 (100)	6 (100)	100 (92)	
Retreated	3 (4)	6 (23)	0 (0)	0 (0)	9 (8)	
BCG						0.86
No	23 (32)	8 (31)	2 (50)	1 (17)	34 (31)	
Yes	20 (27)	10 (38)	1 (25)	2 (33)	33 (30)	
Unknown	30 (41)	8 (31)	1 (25)	3 (50)	42 (39)	
Sputum grading						0.01
Scanty	0 (0)	2 (8)	0 (0)	0 (0)	2 (2)	
1 +	10 (14)	9 (35)	1 (25)	2 (33)	22 (20)	
2 +	16 (22)	6 (23)	1 (25)	3 (50)	26 (24)	
3 +	47 (64)	9 (35)	2 (50)	1 (17)	59 (54)	
Site of TB						0.81
Lung	69 (95)	24 (92)	4 (100)	6 (100)	103 (94)	
Lung plus other	4 (5)	2 (8)	0 (0)	0 (0)	6 (6)	

L: lineage; HIV: human immunodeficiency virus; BCG: Bacillus Calmette–Guérin vaccine; * Fisher’s exact test.

**Table 5:** The number (%) of MTB Isolates with Different Drug Susceptibility Profile Belonging Various Major Lineages.

	L1 n (%)	L2 n (%)	L3 n (%)	L4 n (%)	Total n (%)	*p* Value*
Total	39	19	2	3	63	
Isoniazid						0.92
Sensitive	31 (79)	14 (74)	2 (100)	3 (100)	50 (79)	
Resistant	8 (21)	5 (26)	0 (0)	0 (0)	13 (21)	
Rifampicin						0.24
Sensitive	39 (100)	17 (89)	2 (100)	3 (100)	61 (97)	
Resistant	0 (0)	2 (11)	0 (0)	0 (0)	2 (3)	
Pyrazinamide						1
Sensitive	38 (97)	18 (95)	2 (100)	3 (100)	61 (97)	
Resistant	1 (3)	1 (5)	0 (0)	0 (0)	2 (3)	
Ethambutol						0.71
Sensitive	37 (95)	17 (89)	2 (100)	3 (100)	59 (94)	
Resistant	2 (5)	2 (11)	0 (0)	0 (0)	4 (6)	
Streptomycin						0.04
Sensitive	38 (97)	14 (74)	2 (100)	3 (100)	57 (90)	
Resistant	1 (3)	5 (26)	0 (0)	0 (0)	6 (10)	
PAS						0.38
Sensitive	39 (100)	18 (95)	2 (100)	3 (100)	62 (98)	
Resistant	0 (0)	1 (5)	0 (0)	0 (0)	1 (2)	
Ethionamide						0.82
Sensitive	35 (90)	16 (84)	2 (100)	3 (100)	56 (89)	
Resistant	4 (10)	3 (16)	0 (0)	0 (0)	7 (11)	
At least one TB drug-resistant						0.42
Sensitive	28 (72)	11 (58)	2 (100)	3 (100)	44 (70)	
Resistant	11 (28)	8 (42)	0 (0)	0 (0)	19 (30)	

L: lineage; PAS: para-aminosalicylic acid; * Fisher’s exact test.

**Table 6:** Identified Variants Associated with Drug Resistance in the Genomes of the 19 Clinical *Mycobacterium tuberculosis* Complex Isolates.

Sub lineage	Rif	INH	PZA	ETB	STM	FQ	AM	ETO	PAS	CS
2_2_1_M_ Asian_ African_2	*rpoB*_p.Ser450Leu	*katG*_p.Ser315Thr	*pncA*_p.Gly97Asp	*embB*_p.Met306Val	*rpsL*_p.Lys43Arg	-	-	-	-	-
2_2_2	*rpoB*_p.His445Asp	*katG*_p.Ser315Thr	-	*embB*_p.Met306Ile	*rpsL*_p.Lys43Arg	-	-	-	-	-
1_1_2_2	-	*katG*_p.Ser315Thr	-	*embB*_p.Asp328Gly	*rrs*_r.514a > c	-	-	-	-	-
2_2_1_Modern	-	*katG*_p.Ser315Thr	-	-	*rrs*_r.517c > t	-	-	-	-	-
1_2_1	-	*katG*_p.Ser315Thr	-	-	-	-	-	*fabG1*_c.-15C > T	-	-
2_2_1_M_ Asian_ African_2	-	*fabG1*_c.-15C > T	-	-	-	-	-	*fabG1*_c.-15C > T	-	-
1_1_3_1	-	*fabG1*_c.-15C > T	-	-	-	-	-	*fabG1*_c.-15C > T	-	-
2_2_1_Modern	-	*katG*_p.Ser315Thr	-	-	*rpsL*_p.Lys43Arg	-	-	-	-	-
1_1_2_2	-	-	*panD*_p.Ile49Val	-	-	-	-	*ethA*_c.1054_1054del	-	-
2_2_1_Modern	-	-	-	-	*rpsL*_p.Lys43Arg	-	-	*ethR*_p.Ala95Thr	-	-
1_1_1_8	-	*katG*_p.Ser315Thr	-	-	-	-	-	-	-	-
1_1_3	-	*katG*_p.Ser315Thr	-	-	-	-	-	-	-	-
1_1_3_1	-	*katG*_p.Ser315Thr	-	-	-	-	-	-	-	-
1_2_1_2	-	*katG*_p.Ser315Thr	-	-	-	-	-	-	-	-
1_2_2	-	-	-	-	-	-	-	*ethA*_c.341_341del	-	-
1_1_1	-	*ahpC*_c.-52C > T	-	-	-	-	-	-	-	-
2_2_1_Asia_ Ancestral_3	-	-	-	-	-	-	-	-	*folC*_p.Thr20Pro	-
2_2_1_M_ Asian_ African_2	-	-	-	-	-	-	-	*ethA*_c.24_25insC	-	-
1_2_2	-	-	-	*embB*_p.Phe285Leu	-	-	-	-	-	-

Rif: rifampicin; INH: isoniazid; PZA: pyrazinamide; ETB: ethambutol; STM: streptomycin; FQ: fluoroquinolones; AM: aminoglycosides; ETO: ethionamide; PAS: para-aminosalicylic acid; CS: cycloserine.

**Table 7:** Distribution of MTB lineages in our study compared to other studies.

	Our Study n (%)	Yangon n (%)	Thailand n (%)	Philippines n (%)	Nepal n (%)
**Lineage**	**109**	**72**	**1170**	**178**	**498**
L1	73 (67%)	9 (13%)	480 (41%)	143 (80%)	32 (6%)
L2	26 (23%)	55 (76%)	521 (45%)	2 (1%)	241 (48%)
L3	4 (4%)	4 (5%)	11 (1%)	0	153 (32%)
L4	6 (6%)	4 (5%)	158 (13%)	33 (19%)	72 (14%)

L: lineage.
